# The Potential Role of Inflammation in Modulating Endogenous Hippocampal Neurogenesis After Spinal Cord Injury

**DOI:** 10.3389/fnins.2021.682259

**Published:** 2021-06-18

**Authors:** Arthur Sefiani, Cédric G. Geoffroy

**Affiliations:** Department of Neuroscience and Experimental Therapeutics, College of Medicine, Texas A&M University, Bryan, TX, United States

**Keywords:** neurogenesis, spinal cord injury, inflammation, memory and cognitive impairment, therapeutics

## Abstract

Currently there are approximately 291,000 people suffering from a spinal cord injury (SCI) in the United States. SCI is associated with traumatic changes in mobility and neuralgia, as well as many other long-term chronic health complications, including metabolic disorders, diabetes mellitus, non-alcoholic steatohepatitis, osteoporosis, and elevated inflammatory markers. Due to medical advances, patients with SCI survive much longer than previously. This increase in life expectancy exposes them to novel neurological complications such as memory loss, cognitive decline, depression, and Alzheimer’s disease. In fact, these usually age-associated disorders are more prevalent in people living with SCI. A common factor of these disorders is the reduction in hippocampal neurogenesis. Inflammation, which is elevated after SCI, plays a major role in modulating hippocampal neurogenesis. While there is no clear consensus on the mechanism of the decline in hippocampal neurogenesis and cognition after SCI, we will examine in this review how SCI-induced inflammation could modulate hippocampal neurogenesis and provoke age-associated neurological disorders. Thereafter, we will discuss possible therapeutic options which may mitigate the influence of SCI associated complications on hippocampal neurogenesis.

## Introduction

**FIGURE 1:**
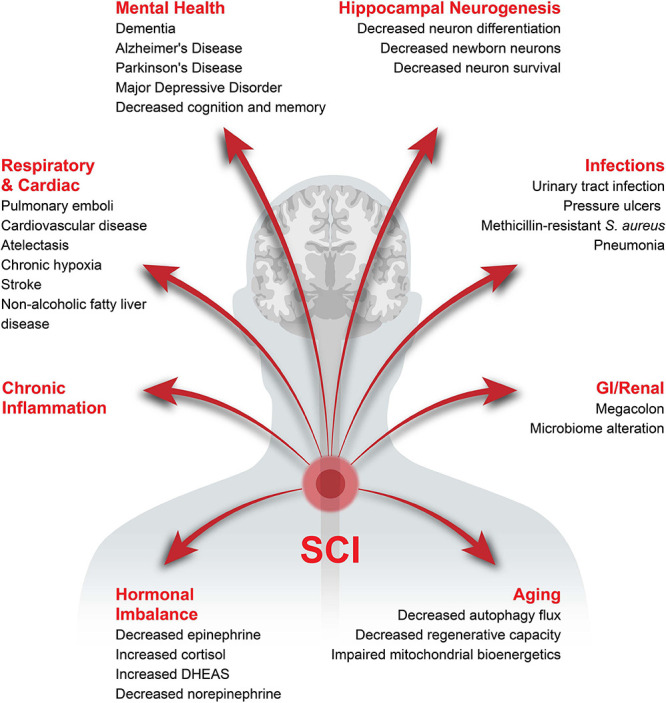
Health complications associated with chronic SCI. The potential health complications developing after SCI have been categorized into major sections with the specific complications in the respective category listed underneath.

Spinal Cord Injury (SCI) is defined as trauma to the spinal cord leading to complete or incomplete injuries and represents the second most common cause of paralysis behind stroke (Armour et al., 2016). According to the National Spinal Cord Injury Statistical Center, there are over 17,000 new cases each year in the United States alone with motor vehicle accidents being the most common cause (Singh et al., 2014), 77.1% of victims being male (Devivo, 2012). The average lifetime medical costs for a SCI victim 25 years of age at time of injury is $1.5-4.7 million depending on severity (Christopher & Dana Reeve Foundation, 2020). Besides locomotion impairment, people living with SCI suffer from a wide variety of health complications, leading to a reduction in life expectancy and quality of life (Figure 1). The location of injury determines pathologies such as loss of respiratory, bowel and bladder control which, along with the severity of trauma, determines the probability of recovery (Alizadeh et al., 2019; Christopher & Dana Reeve Foundation, 2020; Shepherd Center, 2020; SpinalCord.Com, 2020).

One understudied area in the field of SCI is the development of neurological and psychological disorders, cognitive deficits and memory impairments. However, these issues associated with SCI are becoming more apparent since the demographic of the SCI population has shifted in the last few decades. Indeed, the average age at injury is increasing, from 29 in the 1970s to 43 today (National Spinal Cord Injury Statistical Center, 2019), with a peak in the 17-29 group and a second in an older population (> 70 years) (James and Alice, 2019). Additionally, the average age of persons living with SCI is continuously increasing, with and average age of 48 and around 80% being over 40 years old (Christopher & Dana Reeve Foundation, 2021). The rates of incidence of neurological and psychological disorders is increased in people with chronic SCI (Anderson et al., 2007; Huang et al., 2017). Similarly, people with SCI have a 13-fold higher probability of cognitive impairments (Craig et al., 2017), specifically, an impaired ability to learn new tasks and create new memories with a decrease in information processing speed, verbal fluency (Chiaravalloti et al., 2020), visual memory, and perceptual reasoning (Macciocchi et al., 2013). The reasons behind these cognitive declines after SCI are unknown. Memory and cognitive impairments are associated with the reduction of hippocampal neurogenesis in non-SCI models (Bruel-Jungerman et al., 2005; Aimone et al., 2009; Costa et al., 2015). Therefore, one hypothesis is that the cognitive deficits and memory decline in people with SCI, compared to healthy age-matched controls, might be due to decreased hippocampal neurogenesis. In this review, we will focus on the impact of SCI on hippocampal neurogenesis, while it is noteworthy that other age-related processes might also induce these cognitive changes such as loss of both gray and white matter (Resnick et al., 2003) and reduction in synaptic density (Terry and Katzman, 2001).

**FIGURE 2:**
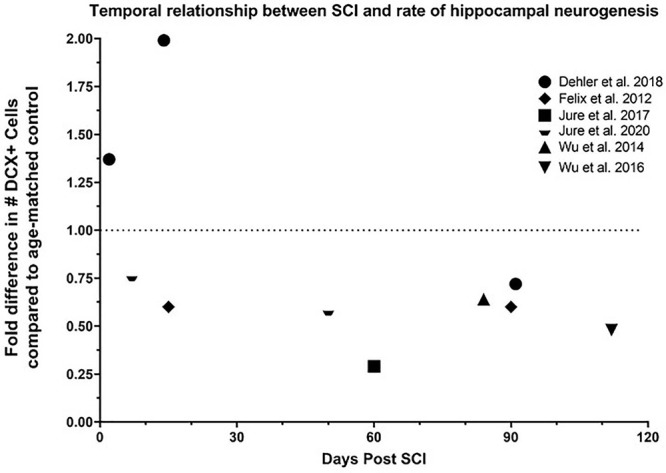
Temporal relationship between SCI and rate of hippocampal neurogenesis. Dehler et al. presented a 37% increase 2 days post injury (dpi), 99% increase 14 dpi, and 28% decrease 91 dpi in the number of DCX+ neurons in the dentate gyrus in a T8 80% transection model on 12-week-old mice (Dehler et al., 2018). Felix et al. reported a 40% decrease 15 and 90 dpi in the number of BrdU+/DCX+ neurons in the dentate gyrus in a C2 left transection in 16-week-old rats (Felix et al., 2012). Jure et al. illustrated a 71% decrease in the number of DCX+ neurons in the dentate gyrus 60 days after severe T8 compression on 8-week-old rats (Jure et al., 2017), and 24% and 42% decrease in the number of DCX+ neurons in the dentate gyrus 7 and 50 days after moderate T8 compression, respectively, on 8-week-old mice. Wu et al. demonstrated a 36% and 52% reduction in the number of DCX+ neurons in the dentate gyrus 84 and 112 days after a moderate T9 contusion in 20-26g mice, respectively (Wu et al., 2014, 2016).

There are only a handful of reports associated SCI with the reduction of hippocampal neurogenesis in pre-clinical models (Figure 2 and Table 1). Some reports associated inflammation with the reduction of neurogenesis while the direct causative effect has not been determined (Wu et al., 2014). However, reduction in hippocampal neurogenesis and SCI have a common denominator, inflammation. Indeed, in non-SCI models, inflammatory response is heavily involved in reducing hippocampal neurogenesis (Wu et al., 2013). Induced inflammatory response after SCI is a well-studied field in both the acute and chronic phases (Sato et al., 2012; Herman et al., 2018). How these SCI-induced inflammatory responses might alter hippocampal neurogenesis is unknown.

**TABLE 1:** Relationship between spinal cord injury (SCI) and rate of hippocampal neurogenesis from different sources.

**Source**	**SCI Type**	**Animal**	**Age**	**Effect (DPI)**
*Dehler et al., 2018*	*T8 80% Transection*	*Female Mouse C57BL/6N*	*12-week-old*	+ *37% (2)* +*99% (14) −28% (91)*
*Felix et al., 2012*	*C2 left Transection*	*Female Sprague–Dawley Rat*	*16-week-old*	*−40% (15) −40% (90)*
*Jure et al., 2017*	*Severe T8 Compression*	*Male Sprague–Dawley Rat*	*8-week-old*	*−71% (60)*
*Jure et al., 2020*	*Moderate T8 Compression*	*Male Mouse*	*8-week-old*	*−24% (7) −42% (50)*
*Wu et al., 2014*	*Moderate T9 Contusion*	*Male Mouse C57BL/6J*	*22-26g Adult*	*−36% (84)*
*Wu et al., 2016*	*Moderate T9 Contusion*	*Male Mouse C57BL/6J*	*20-25g Adult*	*−52% (112)*

*The source of each publication, type of SCI model used, the species, age, and sex of the subject is noted to compare to changes in hippocampal neurogenesis. The Effect (DPI) is expressed as the percent increase/decrease in the number of DCX + cells in the dentate gyrus of subjects with SCI compared to age-matched controls with the respective day post injury (DPI) the quantification took place.*

**FIGURE 3:**
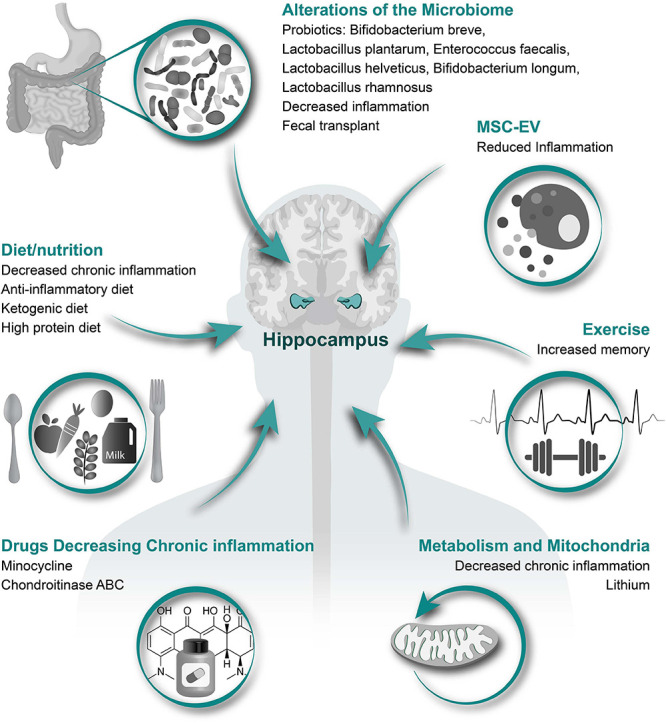
Therapeutic options to increase hippocampal neurogenesis. Illustrated here are several different categories of therapeutic options that have potential to alleviate the SCI induced reduction in hippocampal neurogenesis with specific treatment options and potential affects listed underneath each category.

In this review, we will first discuss the associations between hippocampal neurogenesis and neurological diseases, and neurogenesis after SCI. Next, we will explain how acute inflammation after SCI has potential to either reduce or possibly enhance neurogenesis. A major part of this review will be describing how SCI-induced chronic inflammation might reduce hippocampal neurogenesis and induce cognitive and memory disorders, addressing changes in inflammation associated with aging and several health-issues induced by SCI, including infection, gastrointestinal dysfunctions, respiratory complications and hormonal imbalance (Figure 1 and Table 2). We will finish by proposing therapeutic options that might be of interest to increase hippocampal neurogenesis via the reduction in local and systemic inflammation (Figure 3). It is of high interest to better understand how SCI can reduce hippocampal neurogenesis and contributes to the increased prevalence of cognitive decline and neurodegenerative diseases. Only then will it be conceivable to use the modulation of hippocampal neurogenesis as a tool to reduce the rate of cognitive decline, memory loss, and associated neurodegenerative diseases people with SCI suffer.

**TABLE 2:** Factors, signaling molecules, hormones and activities following SCI that impact hippocampal neurogenesis and inflammation in some cases.

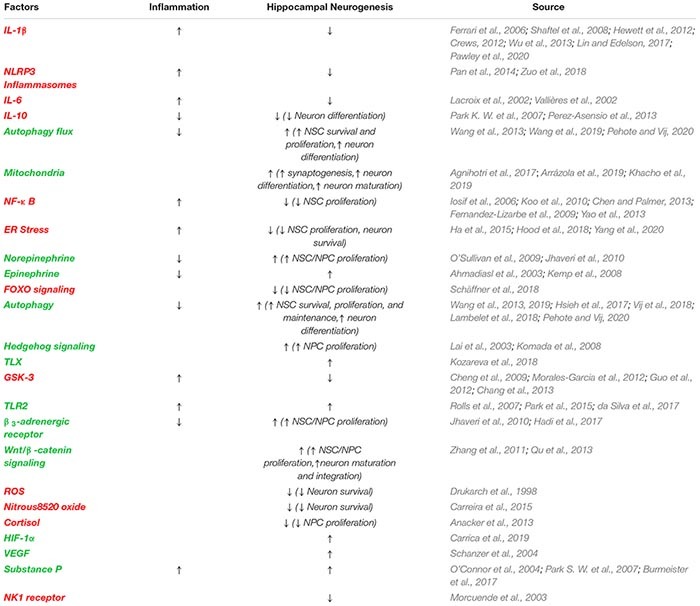

*The arrows show changes (↑: increase; ↓: decrease) in inflammation and hippocampal neurogenesis. The factors are labeled in respect to their positive (green) or negative (red) influence on hippocampal neurogenesis. If the specific process affected during hippocampal neurogenesis is known, it is mentioned in parentheses.*

## Associations Between Hippocampal Neurogenesis and Neuropathogenesis

Hippocampal neurogenesis occurs in a region called the subgranular zone (SGZ), producing granule cells in the dentate gyrus (Toni and Schinder, 2015) throughout a human’s lifetime (Eriksson et al., 1998; Boldrini et al., 2018; Moreno-Jimenez et al., 2019), although its presence in humans during adulthood has been the center of much controversy for decades (Lima and Gomes-Leal, 2019) due to the substantial reduction of hippocampal neurogenesis with aging (Sorrells et al., 2018) causing great difficulty in finding and analyzing in aged subjects (Moreno-Jiménez et al., 2021). Furthermore, the doublecortin (DCX) used to label immature neurons is also expressed in adult entorhinal, amygdala, and parahippocampal neurons (Sorrells et al., 2021) which may artificially increase the calculated rate of neurogenesis. Hippocampal neurogenesis is the process of which neural stem cell (NSC, radial glia-like type 1 cells) transitioning through transiently amplifying neural progenitor cells (NPC) types 2 and 3, transform into the early postmitotic maturation phase where it is decided if the newborn neurons will go through apoptosis or mature. 30–70% of newborn hippocampal neurons and neuroblasts undergo apoptosis within the first month (Dayer et al., 2003; Ryu et al., 2016). Notably, the largest population of newborn cells undergoing apoptosis in the hippocampus are amplifying NPC that are transitioning into neuroblasts (Sierra et al., 2010). Next, the neurons undergo neuritogenesis and synaptogenesis as they mature through the late postmitotic maturation phase, migrate into the inner granule cell layer as they form complex dendritic processes to the molecular layer and an elongated axon connecting to the CA3, integrating into the neural network (Song et al., 2012; Kempermann et al., 2015).

### Roles of Hippocampal Neurogenesis

The role of hippocampal neurogenesis in memory and learning is prominent. There is evidence implicating hippocampal neurogenesis’ role in influencing the rate of recovery of memory capacity, maintaining memory capacity throughout adulthood (Alam et al., 2018) and long-term memory formation in rats (Bruel-Jungerman et al., 2005) and new memories (Aimone et al., 2009). Hippocampal neurogenesis in mice has been credited with providing cognitive flexibility (Burghardt et al., 2012; Anacker and Hen, 2017) and pattern separation (Sahay et al., 2011). The rate of hippocampal neurogenesis is positively correlated with the recovery of memory capacity (Alam et al., 2018) and spatial memory performance in rats (Drapeau et al., 2003), and ability to retain new memory in mice (Kempermann and Gage, 2002). Conditional ablation of hippocampus neurogenesis in APPswe/PS1ΔE9 transgenic mice induced deficits in pattern separation and contextual fear conditioning (Hollands et al., 2017). Increasing hippocampal neurogenesis enhances pattern separation abilities in mice (Sahay et al., 2011). Focal X-irradiation of hippocampal neurogenesis in mice lead to a reduction in contextual fear conditioning with no effect on spatial memory (Saxe et al., 2006). Clelland et al. demonstrated with 2 distinctive methods, X-irradiation and genetic manipulation, that ablation of hippocampal neurogenesis in mice decreases spatial discrimination for only similar spatial locations but not distinct spatial locations (Clelland et al., 2009). Notably, radiation-induced brain injuries provoke a strong inflammatory response in the brain (Greene-Schloesser et al., 2013) which may impact surrounding regions as well.

There are many associations between improving hippocampal neurogenesis, either through exercise (van Praag et al., 1999; Trinchero et al., 2019) or estrogen (Tanapat et al., 1999), and increase in cognitive performance and memory (Daniel et al., 1997; Packard and Teather, 1997; Chang and Etnier, 2009). Increasing hippocampal neurogenesis in mice lead to increased inhibitory activity in the trisynaptic circuit, an acute increase in spatial memory and allocentric navigation, and maintained procedural and contextual memories in aged mice (Berdugo-Vega et al., 2020). The process of learning directly induces a 32% increase in neurogenesis (Leuner et al., 2004) and a 94% increase in number of bromodeoxyuridine (BrdU) + cells in the rat dentate gyrus (Gould et al., 1999) demonstrating a positive correlation between learning and the rate of hippocampal neurogenesis. Some studies suggest the increased prevalence of immature neurons is due to increased new neuron cell survival rather than increased cell proliferation rate (Tashiro et al., 2007; Anderson et al., 2011). Tashiro et al. illustrated increased survival of NeuN + /BrdU + cells in mice born ≤ 3 weeks prior to enrichment with the greatest increase in neurons born 1–2 weeks prior to enrichment (Tashiro et al., 2007). Epp et al. observed a higher number of BrdU + cells in rats regardless of learning type when BrdU was injected 6-10 days prior to learning, and an increase in the percentage of NeuN/BrdU + cells when BrdU was injected 1–5 days prior to learning, compared to rats that did not undergo learning. Since these neurons were born before learning took place, the data suggest an increase in neuron survival rather than in neurogenesis. Learning type also matters, as place-trained rats had higher number of BrdU + cells in the granular cell layer when BrdU was injected 6–10 days prior to learning compared to cue-trained rats (Epp et al., 2007). Further experimentation is needed to determine how specific learning activities affect the survival of each cell type and if the mitigation of increased neuron survival will impact cognitive processes. Due to the difficulties of measuring changes in hippocampal neurogenesis in live subjects, experimentation is usually done on small rodents. There is a possibility that the mechanism of learning and memory formation in humans differs significantly.

### Connection Between SCI, Neurological Diseases and Hippocampal Neurogenesis

Mental wellbeing is an important factor for healthy aging; mental disorders decrease life expectancy by 10.1 years on average (Insel, 2015). People living with SCI are 2.14 times as likely to develop dementia (Huang et al., 2017), 1.71 times as likely to develop Alzheimer’s disease (Yeh et al., 2018), and 1.65 times as likely to develop Parkinson’s disease (Yeh et al., 2016). Unfortunately, people living with SCI are much more likely to commit suicide with reports in England showing a 5-fold increase in per capita suicide rates (Savic et al., 2018). Furthermore, 28% of rats with T12 contusion SCI suffered from depression (Brakel et al., 2019) which was associated with increased serum levels of interleukin (IL)-6 and IL-1α before and IL-6 and tumor necrosis factor (TNF)-α after injury (Brakel et al., 2021). These findings imply predisposition to depression even before injury occurs if systemic increases in inflammation induces depression (Lee and Giuliani, 2019). 49.3% of persons with SCI suffer mild to severe depression (Khazaeipour et al., 2015), 4.3-fold greater than the national average for Major Depressive Disorder (Pedersen et al., 2014). Mental health is also dependent on SCI severity; individuals with high severity SCI are 1.85 times as likely to develop depression than those suffering from less severe SCI (Lim et al., 2017). Furthermore, those suffering from urinary incontinence have a 78% higher probability of developing schizophrenia (Hsu et al., 2017); urinary incontinence is positively correlated with increasing severity of SCI (Elmelund et al., 2018). Therefore, SCI-induced mental disorders are common disruptions of healthy aging and longevity. However, a direct link between SCI and the development of any of these neurological diseases is missing. We propose here that this link might be the reduction in hippocampal neurogenesis.

Indeed, many neuropathological disorders leading to cognitive and memory impairments are associated with impaired hippocampal neurogenesis (Costa et al., 2015; Han et al., 2016). In Alzheimer’s disease, there is a significant reduction in hippocampal neurogenesis (Haughey et al., 2002), the results of several mechanisms, including β-amyloid (Aβ) plaque (Haughey et al., 2002) and Aβ42 assemblies (Sun et al., 2009). Parkinson’s disease, the second most common neurodegenerative disease (Bertram and Tanzi, 2005) which is also associated with cognitive impairment in a subset of patients (Watson and Leverenz, 2010), can be caused by α-Synuclein (α-syn) accumulation around dopaminergic neurons (Masliah et al., 2000). Not only does overexpression and aberrant α-syn accumulation perturb hippocampal neurogenesis (Winner et al., 2012; Winner and Winkler, 2015), but passive immunization against α-syn reverses cognitive and memory impairments (Masliah et al., 2011) which are hippocampus associated functions. The size of the hippocampus of humans with schizophrenia are reduced (Steen et al., 2006) with a reduction in the number of Ki67 + cells, although no changes in the number of NeuN + were noted (Reif et al., 2006; Allen et al., 2016). Rats with depression had decreased number of DCX + cells in the suprapyramidal blade of the dentate gyrus (Brakel et al., 2021). Increasing hippocampal neurogenesis through genetic manipulation was able to rescue mice from depressive like behaviors induced by chronic corticosterone administration (Hill et al., 2015).

Inflammation and infections, which have potential in inducing neurodegenerative diseases such as Parkinson’s disease (Caggiu et al., 2019), also influence hippocampal neurogenesis by reducing the number of BrdU/NeuN double positive cells in the dentate gyrus of rats (Ekdahl et al., 2003). In these neuropathologies, some of the patient’s symptoms, such as impaired cognitive function and memory loss, may be due to the decreased levels of hippocampal neurogenesis. Thus, it is hypothesized that reversing changes to hippocampal neurogenesis has clinical significance in reversing cognitive decline and memory loss (Peng and Bonaguidi, 2018). Hippocampal neurogenesis is also implicated in helping preserve the dentate gyrus after a prion infection in mice (Gomez-Nicola et al., 2014). Nonetheless, the direct mechanism of cognitive decline induced by neurodegenerative diseases and the direct influence of hippocampal neurogenesis on mental and neurological wellbeing has yet to be elucidated.

### Hippocampal Neurogenesis After SCI

Only a handful of studies have assessed the impact of SCI on hippocampal neurogenesis (Figure 3 and Table 1). SCI-induced modulation of hippocampal neurogenesis is time-, severity-, age- and model-dependent. The rate of hippocampal neurogenesis (assessed by the quantification of either DCX + or DCX/BrdU + cells in the dentate gyrus) significantly declines at 7 days post injury (dpi), 50 dpi (Jure et al., 2020), 60 dpi (Jure et al., 2017), 84 dpi (Wu et al., 2014), and 112 dpi (Wu et al., 2016) after moderate to severe thoracic SCI while the mild SCI model in these studies showed no significant changes. Felix et al. using a left transection SCI model found decreased hippocampal neurogenesis just 15 dpi (in coherence with Jure et al.) which remained significantly decreased during the entire 3-month study after cervical transection (Felix et al., 2012). This is contradictory to another study which reported a significant increase in DCX + /BrdU + cells 2 and 14 dpi in an 80% thoracic transection model. This was accompanied with a significant decline in hippocampal neurogenesis (28% below baseline) at a chronic time point (91 dpi) (Dehler et al., 2018). Major differences between studies were the location of injury (cervical vs thoracic) and animal species used (mouse vs. rat). The location of injury does generally impact both motor control and organ dysfunction differently (Alizadeh et al., 2019) which might also influence hippocampal neurogenesis differently. Subject species might also impact neurogenesis differently since rats and mice have different inflammatory reactions to SCI. Mice have delayed T-cell entry and less, if any, influx of dendritic cells into the injury site compared to rats (Sroga et al., 2003). Dendritic cells are antigen-presenting cells that initiate adaptive immune function (Martin-Gayo and Yu, 2019). Nevertheless, Jure et al. confirmed Felix’s findings of decreased hippocampal neurogenesis at 7 dpi using the same species and location as Dehler et al. (mice, T8); although, unlike Felix and Dehler, Jure et al. used a compression model which may impact hippocampal neurogenesis and systemic inflammation differently. Transection and contusion SCI models can invoke different inflammatory changes. After transection, there is damage to the meninges which support neuroinflammation (Rua and McGavern, 2018). Leaking cerebral spinal fluid may lead to the reduction in microglia around the injury site of rats with spinal transections (Watanabe et al., 1999). Contusions also induce greater rostrocaudal spread of pathologies (Hausmann, 2003). Furthermore, Jure observed no change and Wu observed a smaller decrease in neurogenesis in less severe (mild) SCI models. We hypothesized that milder SCI perturb hippocampal neurogenesis less than severe SCI due to being associated with less severe inflammation and smaller inflammatory responses compared to severe injuries (Yang et al., 2005). BrdU injections for the last 4 weeks of a 4-month (using a T9 contusion model) demonstrated a significant decrease in BrdU/NeuN + and%BrdU/NeuN + cells and a downward trend in %BrdU/DCX + cells. These data are implicative of both a decrease in neuron differentiation and survival of newborn neurons during chronic SCI (Wu et al., 2016). Altogether, injury type and severity are likely to influence neurogenesis patterns. It may explain why the 2 largest decreases in hippocampal neurogenesis are seen in studies using the compression and contusion model (Wu et al., 2016; Jure et al., 2017). Therefore, a more careful and systemic characterization of the impact of SCI on neurogenesis in relationship to time after injury, severity of injury, age, sex, subject specie, and injury type is needed to further elucidate the discrepancies found in these studies.

## Relation Between SCI, Acute Inflammation, and Hippocampal Neurogenesis

Spinal Cord Injury activates various inflammatory pathways. Throughout recovery, there are dynamic changes in both the severity and polarization of inflammation. These modulations have potential to influence hippocampal neurogenesis and associated processes.

### Acute Inflammatory Changes in the Spinal Cord Following SCI

Spinal Cord Injury leads to necrosis, activating the immune system (van Den Hauwe et al., 2020) leading to a rapid increase in macrophages, activated microglia, and CD45 + leukocytes at the injury site (Okada, 2016). Within 30 min of SCI, IL-1β and TNF-α signaling across the entire spinal cord increases followed by IL-6 expression increasing in both spinal astrocytes and microglia and leukemia inhibitory factor (LIF) expression increasing only in spinal astrocytes at the injury site within 3 h (Pineau and Lacroix, 2007; Sutherland et al., 2017). Since the permeability of the blood spinal cord barrier is significantly elevated immediately after SCI (Cohen et al., 2009) while the blood brain barrier permeability is rapidly increased for the first 24 h after SCI (Donnelly and Popovich, 2008), there is potential for inflammatory biological agents to be quickly transported from the injury site to the hippocampus through the cardiovascular system. Another possible mechanism for rapid systemic inflammatory changes following SCI is changes in the extracellular vesicle (EV) profile being released into the bloodstream (Dutta et al., 2021). After SCI in mice, there is an increased proportion of CD81 + EVs found in the plasma for the first 7 dpi. Intracranial injections of CD81 + EVs collected after SCI increased astrocyte IL-1β and IL-1α cytokine levels and astrocyte reactivity gene expressions suggesting CD81 + EVs have potential to modulate the reactivity of astrocytes even in regions distant from the injury site (Khan et al., 2021). CD81 has been implicated in increasing the migration of activated T lymphocytes (Hasezaki et al., 2020) and inducing some of the neurological pathologies associated with experimental autoimmune encephalomyelitis (EAE) (Dijkstra et al., 2008). To determine the direct influence of inflammatory cytokines on recovery, IL-6 was injected into the lesion core causing a 6-fold and 2-fold increase in neutrophil and microglial presence, respectively, while increasing the size of the damaged lesion core (Lacroix et al., 2002). Therefore, proper modulation of the inflammatory system is necessary to mitigate tissue trauma.

In response to SCI, p38 MAPK is activated in reactive spinal microglia producing IL-1, IL-6, TNF-α, and prostaglandin (PD)E_2_ (Ji and Suter, 2007; Sato et al., 2012). Notably, microglia also produce anti-inflammatory cytokines like IL-10 (Park K. W. et al., 2007). The physical penetration of foreign objects into the spinal cord increases the probability of a pathogenic infection. Lipopolysaccharides (LPS) from those pathogens induce signal transducer and activator of transcription (STAT)1 signaling which promotes pro-inflammatory microglial phenotype (Liu et al., 2012) and stimulates the release of reactive oxygen species (ROS) and pro-inflammatory cytokines from microglia (Butturini et al., 2019). COX-1 expressing spinal microglia also increased for up to 4 weeks after SCI (Schwab et al., 2000a). Although unclear how COX-1 can impact persons with SCI, COX-1 is highly associated with other neurological disorders including neuroinflammation (Choi et al., 2008; Aid et al., 2010; Choi et al., 2010), Alzheimer’s disease (Yermakova et al., 1999), and ischemia (Schwab et al., 2000b). Microglia are vital neuroprotective components of our central nervous system (CNS) (Streit, 2005), preventing neuron and oligodendrocyte death (Bellver-Landete et al., 2019), stripping synapses (Chen and Trapp, 2016), and increasing spinal cord repairs (Fu et al., 2020; Li et al., 2020a). Microglia can also secrete Interferon (IFN)-β to remove axon and myelin debris from the injury site (Kocur et al., 2015), increase axon regenerative potential, and improve functional locomotor recovery (Tanaka et al., 2009).

After SCI, there is an elevated level of astrocyte activation for at least the first week after injury (Schnell et al., 1999). These reactive astrocytes in the lesion penumbra, long thought to inhibit axon regeneration (Rudge and Silver, 1990) have also been shown to improve healing and locomotor functional recover while acting as a neuroprotectant (Bush et al., 1999; Faulkner et al., 2004). Astrocytes also protect and maintain the blood brain barrier (Cabezas et al., 2014) which becomes significantly more permeable after SCI (Donnelly and Popovich, 2008). Reactive astrocytes become scar-forming (Okada et al., 2018) and produce TNF-α, IL-1β, interferon gamma-induced protein (IP)-10, macrophage inflammatory protein (MIP)-1α, and C-C chemokine ligand (CCL)5 (Choi et al., 2014). Overall, reactive astrocytes are essential in recovery after SCI, mitigating cell death and loss of locomotor function (Faulkner et al., 2004).

### SCI-Induced Acute Inflammation May Modulate Hippocampal Neurogenesis

There are several inflammatory molecules that are altered acutely after SCI which could positively or negatively impact hippocampal neurogenesis. Unless the changes were analyzed in the hippocampus, it is unknown if changes only occurring in the spinal cord can impact hippocampal processes. Quickly after SCI, several pro-inflammatory molecules, such as TNF-α and IL-1β, are locally secreted in the spinal cord. These factors are known to reduce hippocampal neurogenesis in non-SCI settings (Lan et al., 2012; Wu et al., 2013; Donzis and Tronson, 2014). Jure et al. reported a reduction of newborn hippocampal neurons during the acute stages of SCI, which is associated with increased astrocytic and microglial activation in the hippocampus (Felix et al., 2012; Jure et al., 2020). At 8 dpi, IL-6 expression is elevated in the hippocampus, although, TNF-α, iNOS, and CD68 expressions are not significantly increased (Wu et al., 2014). Therefore, SCI has potential to directly alter cytokine levels in the hippocampus. SCI also induces a significant increase in glucocorticoid expression at the injury site during the first 24 h after injury (Yan et al., 1999). Glucocorticoid expression in the hippocampus, although dose dependent, can reduce NPC proliferation and DCX + neuroblasts while favoring astrocyte differentiation (Anacker et al., 2013). Therefore, it is tempting to speculate that SCI-induced glucocorticoids might directly reduce neurogenesis in the hippocampus. Nevertheless, further experimentation is required to determine the changes in both hippocampal glucocorticoid and cytokine levels immediately after SCI and how these SCI-induced changes directly impact neurogenesis during the acute stages of SCI.

On the other hand, Dehler et al. observed a significant increase in neurogenesis 7 days post SCI (Dehler et al., 2018). Several factors secreted at the lesion site could participate in such increase. Retinoic acid is elevated in the spinal cord 4–14 days post SCI with its decline 21 days post injury (Schrage et al., 2006), and has been shown to increase NSC and NPC proliferation through the activation of hypoxia-inducible factor (HIF)-1α (Mishra et al., 2018). HIF-1α is required for Notch signal activation (Li J. et al., 2018), learning and hippocampal neurogenesis (Carrica et al., 2019). Interestingly, HIF-1α is significantly increased in the spinal cord within 12-24 hours post injury, reaching maximal levels 3-7 days post SCI with a gradual decrease thereafter (Ju et al., 2002; Xiaowei et al., 2006). Vascular endothelial growth factor (VEGF), which is induced by HIF-1α (Lin et al., 2004) and upregulated alongside HIF-1α after SCI (Chen et al., 2017), also stimulates hippocampal neurogenesis (Schanzer et al., 2004) and even reverses age-associated decline in cognition (Han et al., 2017) and hippocampal neurogenesis (Licht et al., 2016). Therefore, one mechanism of increased hippocampal neurogenesis immediately after SCI may be through the increase of VEGF expression following the retinoic acid-induced activation of the HIF-1α, which would activate neurogenesis away from the injury site. Alternatively, retinoic acid might travel to the hippocampus and directly activate HIF-1α in the hippocampal NSC/NPCs.

To the contrary of many inflammatory molecules increased after SCI, substance P, typically secreted by inflammatory cells (O’Connor et al., 2004), is drastically decreased in the spinal cord during the first 4 weeks after injury (Leonard et al., 2014). Substance P activates neurokinin (NK)1 receptor (Schank and Heilig, 2017), and NK1 receptor deletion increases both hippocampal neurogenesis and BDNF levels (Morcuende et al., 2003). Substance P injections into lateral ventricles increased the number of DCX + neurons in the hippocampus by 12% (Park S. W. et al., 2007). Substance P also mediates the LPS-induced production of IL-6 from astrocytes (Burmeister et al., 2017) and has pro-inflammatory effects (O’Connor et al., 2004) which has potential to decrease hippocampal neurogenesis. Altogether, these data suggest the potential role of substance P and NK1 receptor in participating in the modulation of neurogenesis. How the reduction of substance P at the injury site can be linked to acute changes in hippocampal neurogenesis after SCI remains to be determined.

We want to emphasize that the potential beneficial effects of acute inflammation after SCI on neurogenesis are under debate. While it is conceivable that this early increase in neurogenesis is a potential attempt to protect from a future decline in neurogenesis, increasing cell proliferation in the hippocampus immediately after injury will not be of clinical interest if the newborn neurons are not capable of surviving, maturing, and integrating into the hippocampal network. A possible therapeutic avenue may be to mimic the neurogenesis promoting processes induced by acute inflammation mentioned above during the chronic injury stages of SCI. It is hypothesized that the beneficial impact of acute inflammation is time dependent. Therefore, prolonged exposures to such systemic changes must be further elucidated.

## SCI-Induced Chronic Inflammation Neuropathologies and Reduction of Hippocampal Neurogenesis

While the exact acute effects of SCI, and presumably of acute inflammation, on hippocampal neurogenesis is still under debate, all the studies agree that neurogenesis is severely reduced in chronic models of SCI. Aberrant immune behavior in chronic SCI leads to autoimmunity, chronic inflammation and immune deficiency. SCI-induced changes are systemic, almost every organ and system are differently impacted by SCI depending on severity of injury. Ischemia, edema and oxidative damage are increased at the site of injury accumulating further strong inflammatory responses (Carlson et al., 1998). People living with SCI are more susceptible to infections and cardiometabolic dysfunctions (Bauman and Spungen, 2001; Phillips and Krassioukov, 2015). Other dysfunctions commonly occurring after SCI are splenic atrophy, neurogenic bowel dysfunction, inflammation-induced muscle atrophy, osteoporosis, and neurogenic heterotopic ossification (Sun X. et al., 2016). These statistics vary greatly between countries depending on the healthcare and economic status of the country (Singh et al., 2014), 12% of SCI patients die from infections, 21.4% from respiratory disease, and 4.9% from gastrointestinal-renal complications (National Spinal Cord Injury Statistical Center, 2014, 2019). This translates to a 539% increase in infection, 280% increase in respiratory disease, and 139% increase in gastrointestinal-renal related fatalities per capita for people living with SCI relative to the average per capita fatality rates in the United States (National Spinal Cord Injury Statistical Center, 2014; Centers for Disease Control and Prevention, 2017; National Spinal Cord Injury Statistical Center, 2019).

### SCI-Induced Inflammation May Cause Neuropathologies

Several pro-inflammatory and pro-infectious molecules are associated with both SCI and different neuropathologies. Increased IL-1 production as a defense mechanism stimulates β-amyloid precursor protein production (Goldgaber et al., 1989); treatment of hippocampal neurons with IL-6 induces hyperphosphorylation of tau (Quintanilla et al., 2004). Collectively, both IL-1 and IL-6 increase the likelihood of developing Alzheimer’s disease (Iqbal et al., 2010; Hansen et al., 2018; Kinney et al., 2018). IL-1β-induced production of IL-6 (Chen et al., 2005; Trompet et al., 2008) has not only been shown to increase age-associated cognitive decline (Trompet et al., 2008), but also lead to depression (Capuron et al., 2008; Raison and Miller, 2011; Lee and Giuliani, 2019). Elevated IL-6 serum levels are strongly correlated with schizophrenia (Schmitt et al., 2005; Neelamekam et al., 2014). Maternal infections during pregnancy that increased IL-1 β, IL-6, and TNF-α levels in their offspring, hypothesized to be LPS-induced, increased susceptibility to schizophrenia (Gilmore et al., 2004). Similarly, LPS from bacterial species such as *Pseudomonas aeruginosa*, commonly found in people living with SCI, induces Parkinson’s disease in animal models (Dutta et al., 2008; Wang et al., 2015; Huang et al., 2018). Other molecules known to activate inflammatory cytokines, such as chondroitin sulfate proteoglycans (CSPGs) (Stephenson and Yong, 2018), are increased after SCI (Iaci et al., 2007; Dyck and Karimi-Abdolrezaee, 2018), and are involved in Alzheimer’s disease (DeWitt et al., 1993) and neurodegeneration (Galtrey and Fawcett, 2007). SCI-induced inflammation and LPS accumulation may directly cause the increased prevalence of Alzheimer’s disease, Parkinson’s disease, schizophrenia, and other neurological disorders common among persons with SCI. Nonetheless, the direct effects of SCI-induced changes in cytokine levels on hippocampal neurogenesis and neuropathologies have yet to be elucidated. Notably, some cytokines levels are elevated only at the injury site which may not affect hippocampal neurogenesis, although can cause systemic changes since blood vessel permeability is proportional to the level of inflammation (Park et al., 2018) and the permeability of the blood spinal cord barrier is elevated for at least the first 8 weeks after injury (Cohen et al., 2009).

### Relationship Between SCI-Induced Health Complications and Neurogenesis

#### SCI-Induced Infections and Their Effects on Neurogenesis

Spinal Cord Injury patients have much higher rates of infection, in part due to their compromised immune system and their inability to expel excretion fluids (Vyas et al., 2015). Short-term local inflammatory responses should not have prolonged effects on hippocampal neurogenesis, yet systemic infections directly increase both TNF-α and IL-1β levels in the brain (Püntener et al., 2012) which do impact hippocampal processes. *Pseudomonas aeruginosa* associated bloodstream infections were more fatal in SCI patients than in patients on immunosuppressant therapy (Saliba et al., 2017) suggesting SCI has devasting impacts on immune function. Over 74% of SCI patients are infected with methicillin-resistant *Staphylococcus aureus* (MRSA) at one point, a systemic infection that activates GSK-3β leading to increased TNF-α expression, nitric oxide (NO) production (Cheng et al., 2009), NF-κB activation and iNOS expression (Chang et al., 2013). It is estimated that 5–15% of SCI patients will develop pneumonia (Montgomerie, 1997) which is most commonly caused by *Streptococcus pneumonia* (Claudia Antonieta Nieves Prado, 2018). The cell wall of *Streptococcus pneumonia* contains lipoteichoic acid (LTA), which not only induces meningitis, inflammation of brain and spinal cord meninges, but also impairs hippocampal neurogenesis (Hoffmann et al., 2007) by inducing apoptosis of hippocampal NSC and NPC (Hofer et al., 2011). LTA is also found in *Staphylococcus aureus* which interacts with toll-like receptor 2 (TLR2) (Schroder et al., 2003) to activate pro-inflammatory macrophages through a CD14 mediated manner (da Silva et al., 2017). Reduction of pro-inflammatory macrophage activation improved hippocampal neurogenesis (Zhang et al., 2017). Notably, many argue the existence of more than 2 macrophage phenotypes as the role of each phenotype is complex and time-dependent (Sato, 2015). Although TLR2 is required for pro-inflammatory macrophage activation and its over activation affects neural development (Du et al., 2011), TLR2 is also required for hippocampal neurogenesis (Rolls et al., 2007; Park et al., 2015). Therefore, total inhibition of an inflammatory pathway does often come with unattended harmful consequences.

Urinary tract infection (UTI) inducing pathogens are potentially strong contributors to the impaired hippocampal neurogenesis patterns in people with chronic SCI. *Pseudomonas aeruginosa* activates TLR4 and TLR5 in mice (Sun et al., 2010), contributing to the activation of NLR Family CARD Domain Containing 4 (NLRC4) and NLR Family Pyrin Domain Containing 3 (NLRP3) inflammasomes (Lin and Kazmierczak, 2017) similarly to *Klebsiella pneumoniae* (Codo et al., 2018). TLR4 activation directly in the brain has been implicated to inhibit hippocampal neurogenesis in TBI (Ye et al., 2017), development (Rolls et al., 2007) and alcohol addiction (Vetreno et al., 2018) models. Furthermore, systemic inhibition of NLRP3 inflammasomes through a heat shock protein (HSP)90 inhibitor increased BDNF levels and hippocampal neurogenesis (Zuo et al., 2018). Moreover, NLRP3 inflammasomes increase IL-1β production in macrophages and pulmonary inflammation (Kang et al., 2017), increasing susceptibility to further respiratory infections such as pneumonia (Mizgerd, 2018). This suggests the potential for UTI to reduce hippocampal neurogenesis. Indeed, UTI infections will mostly impact local regions around and in urinary tracts and may not affect hippocampal processes. Although, chronic inflammation in one region of the body still has potential to induce systemic changes over time, especially since inflammation increases blood vessel permeability (Park et al., 2018). Persons with SCI are more prone to both local infections, such as UTIs, and systemic infections, such as MRSA. Therefore, the systemic and chronic infections caused by SCI have potential to influence neurological processes, including hippocampal neurogenesis. Further experimentation must be conducted to determine the direct effect of each infection type on hippocampal neurogenesis in both an acute and chronic manner.

#### Role of Cardiometabolic Disorders in Neurogenesis

The prevalence of diabetes mellitus, stroke, and heart disease in person’s with SCI are 3-fold, 3.7-fold, and 2.7-fold higher, respectively, than that of the general population (Lavela et al., 2006; Cragg et al., 2013; Wan and Krassioukov, 2014; Evonuk et al., 2017; Sharif and Hou, 2017). Diabetes in mice increases IL-6, TNFα, GFAP, and Iba-1 expression in the hippocampus (Bhusal et al., 2019). Diabetes impacts hippocampus function, reducing cell proliferation in the dentate gyrus (Stranahan et al., 2008), number of neurons in the hilus (Beauquis et al., 2006) and total number of hippocampal neurons (Bhusal et al., 2019). Diabetes is associated with a reduction in learning efficiency, memory, and spatial cognition in mice (Alvarez et al., 2009; Bhusal et al., 2019) and verbal fluency in women (Kanaya et al., 2004). Stroke (transient global ischemia) in rats, increases astrocyte mediated iNOS production in the hippocampus (Endoh et al., 1994). Stroke in mice impairs hippocampus-dependent spatial memory hypothesized to be caused by aberrant neurogenesis failing to integrate newborn neurons properly (Woitke et al., 2017). Strokes that induced hippocampal infarction in humans led to problems in recognition and long-term memory (Szabo et al., 2009). 7 days after SCI, the death of hepatocytes increase TNF-α and IL-1β expression more than 10-fold in the liver. These changes progress liver dysfunction (Sauerbeck et al., 2015) and non-alcoholic steatohepatitis, leading to metabolic dysfunction during the chronic phases (Goodus and McTigue, 2020). SCI impairs liver function, causing lipid accumulation (Sauerbeck et al., 2015) leading to a 2- fold higher rate of non-alcoholic fatty acid liver disease (NAFLD). NAFLD leads to brain hypoperfusion and reduces both cognitive (Pinçon et al., 2019) and memory capacity (Ross et al., 2012). Those with NAFLD also had diminished testosterone levels (Barbonetti et al., 2016) which is essential for the survival of newborn hippocampal neurons (Spritzer and Galea, 2007)and neuroplasticity (Wainwright et al., 2011). 58.9% of individuals with SCI have low high density lipoprotein cholesterol (HDL-C) (Vichiansiri et al., 2012) which is common in women with severe depression (Ramachandran Pillai et al., 2018) and is associated with memory deficits in humans (Singh-Manoux et al., 2008). Reduction in HDL-C in humans is also associated with reduced gray matter in the parahippocampul region (Ward et al., 2010) which is involved in spatial memory in humans (Bohbot et al., 2015). Cardiometabolic disorders are undoubtedly associated with chronic inflammation (Lopez-Candales et al., 2017) and can influence hippocampal neurogenesis, although, the direct effects of cardiometabolic disorders caused by SCI on neurogenesis have yet to be evaluated.

#### Gastrointestinal-Renal Disorders and Neurogenesis

People living with SCI commonly have major bowel dysfunctions. In fact, 18-73% of them develop megacolon (Harari and Minaker, 2000; Faaborg et al., 2011) which is often associated with inflammatory bowel disease (IBD) and hypothesized to be caused by infections (Hommes et al., 2004; Autenrieth and Baumgart, 2012). IBD quickly increases IL-6, TNFα, IL-10, and IL-1β levels in the hippocampus, resulting in a short-term increase in hippocampal neurogenesis. In the chronic stages, IBD decreases overall proliferation (Zonis et al., 2015), migration, and integration of newborn hippocampal neurons (Gampierakis et al., 2020). Mice with SCI have gut dysbiosis associated with both activation and phenotypical alteration of the immune cells in gut-associated lymphoid tissues (Kigerl et al., 2016). *Clostridium saccharogumia* is depleted in the intestines of rats with SCI (O’Connor et al., 2018). *Clostridium saccharogumia* produces enterolactone in the gut of rats (Woting et al., 2010) which is associated with reducing the risk of cardiovascular disease which might indirectly improve hippocampal neurogenesis. Several studies have analyzed SCI-induced changes in the microbiota (Kigerl et al., 2018; Bannerman et al., 2021; Du et al., 2021), although, it is unclear how these changes can impact hippocampal neurogenesis. Determining the influence of SCI-induced changes to the microbiota and altering it to increase hippocampal neurogenesis is a developing area of research (Jogia and Ruitenberg, 2020; Tang et al., 2021). SCI induces the dysfunction of many peripheral organs which can all participate in the reduction of neurogenesis, although, the direct influence of SCI-induced peripheral organ dysfunction on neurogenesis needs further evaluation.

#### Respiratory Disease and Neurogenesis

Respiratory disease is a major problem in the SCI community; persons with SCI have a 46.9-fold and 37.1-fold higher probability of dying from pulmonary emboli and pneumonia, respectively (DeVivo et al., 1993). Inability to cough can prevent the clearing of contaminants from the lungs, further increasing infection rate. Atelectasis, commonly occuring in SCI patients, decreases areation, coupled with the loss of neurological control of breathing and coughing transforms the lungs into a breeding ground for pathogens causing pnemonia and further activating inflammatory pathways (Berlowitz et al., 2016). Decreased lung and chest wall compliance may cause the reduction in vital capacity seen in SCI patients (Brown et al., 2006) lessening oxygen consumption efficiency by 46% (Manning et al., 1992). Chronic hypoxia, common among SCI patients, degenerates neural cells (Hernandez-Gerez et al., 2019). Persons with SCI have higher rates of sleep apnea (Khuu et al., 2019) and significantly lower oxygen saturation levels (Fuller et al., 2013), which perturbs hippocampal neurogenesis and synaptic plasticity (Khuu et al., 2019). Furthermore, lipogenesis, which depends on oxygen intake, is required for the proliferation of NSC/NPC and hippocampal neurons (Knobloch et al., 2017) and may also contribute to the 69.8% increase in prevalence of metabolic syndrome in adolescents with chronic SCI (Nelson et al., 2007) creating yet another positive feedback loop. It would be interesting to assess how different regimens of intermittent hypoxia, which increases hippocampal HIF-1α levels (Tregub et al., 2020), could be used to enhance hippocampal neurogenesis. Current research shows intermittent, but not chronic, hypoxia can promote NSC/NPC proliferation and increase BDNF expression, hippocampal neurogenesis, and cognition (Zhu et al., 2005; Zhu et al., 2010; Meng et al., 2020).

#### Hormone Imbalance and Neurogenesis

Hormonal imbalance, regardless of the cause, contributes to a dysfunctional immune system and influences hippocampal neurogenesis. Endocrine disorders are common in people with SCI (Bauman et al., 2017; Gater et al., 2019). Epinephrine and norepinephrine are significantly lower in SCI patients (Schmid et al., 1998). Epinephrine counteracts anaphylaxis (Kemp et al., 2008), consequently, the probability of allergic reactions increase, increasing inflammation in the brain (Sarlus et al., 2012). It is hypothesized that the increase in hippocampal neurogenesis after exercise is mediated through epinephrine (Ahmadiasl et al., 2003), therefore, any reduction in epinephrine production may decrease the beneficial effects of exercise (Petersen and Pedersen, 2005). Norepinephrine is required for antibody production (Kohm and Sanders, 1999), therefore, a lack thereof may also relate to a dysfunctional immune system and increased prevalence of infections. Norepinephrine reuptake inhibitors reduce neuroinflammation (O’Sullivan et al., 2009) and stimulate NSC/NPC proliferation meditated by the β_3_-adrenergic receptor (Jhaveri et al., 2010). The stimulation of the β_3_-adrenergic receptor not only inhibits the LPS/TLR4-induced inflammatory response, but also induces anti-inflammatory effects in macrophages (Hadi et al., 2017). Elevated levels of cortisol and dehydroepiandrosterone sulfate (DHEAS) are present during chronic SCI (Campagnolo et al., 1999; Popovich et al., 2001; Prüss et al., 2017). Like many of the other signaling molecules, the effects of both cortisol and DHEAS are dose dependent. DHEAS increases hippocampal plasticity in the CA1 region, spatial learning and memory only at low doses (Diamond, 2004). Low dose treatment of cortisol increased NPC proliferation, reducing DCX + neuroblasts and favoring astrocyte differentiation, while high dose cortisol treatment impairs both NPC proliferation and hedgehog signaling while increasing FoxO3A signaling (Anacker et al., 2013). Sonic hedgehog signaling increases NPC proliferation (Lai et al., 2003) and neurogenesis during development (Komada et al., 2008). FoxO deletion in adults increases NSC/NPC proliferation but impairs the autophagy flux (Schäffner et al., 2018). Therefore, abolishing cortisol levels may induce more inflammatory dysregulation while low cortisol levels seen in healthy adults may still provide the benefits of FoxO-induced autophagy flux. This provides another example of the need to properly modulate signaling pathways rather than eradicate them.

### Compounding Effects of SCI and Aging

Aging is highly associated with reduced hippocampal neurogenesis (Knoth et al., 2010) and cognitive capacities such as memory, attention, and executive cognitive functions (Murman, 2015). SCI increases the prevalence of age-associated disorders, such as neurodegenerative diseases (Huang et al., 2017) and cognitive deficits (Craig et al., 2017). We hypothesize that older individuals with SCI are more susceptible to aberrant immune function leading to greater incidence rates for disorders associated with hippocampal function. With improvement in modern medicine, we are seeing an unprecedented increase in life expectancy; an increase of 5.5 years just from the year 2000 (World Health Organization, 2020). Although there is a notable increase in life expectancy for people living with SCI, the increase in lifespan has not been comparable to the general population (Groah et al., 2012). Chronic inflammatory diseases are increasingly the most common cause for mortality in the overall population (Remington and Brownson, 2011). Aging with chronic inflammation, as seen in persons with SCI, decreases our ability to regenerate damaged tissue, increasing susceptibility to fatal infections and injuries (Xia et al., 2016). Healthy aging is usually associated with elevated IL-10 and reduced IL-6 levels; however, people requiring increased resistance to infections, such as those with SCI, generally have elevated IL-6 and decreased IL-10 expression instead (Caruso et al., 2004). When comparing healthy uninjured ∼ 7-, ∼ 13-, and ∼23-month-old rats, no significant changes were found in the expression of TNF-α, CXCL10, VEGF, IL-1α, IL-1β, and IL-2/4/6/10/17/18 hippocampal cytokine levels (Scheinert et al., 2015). Therefore, increased pro-inflammatory cytokines production seen in those suffering from chronic SCI is likely directly induced by SCI rather than aging itself. Furthermore, age diminishes microglial sensitivity to IL-4, a pleiotropic anti-inflammatory cytokine associated with polarization toward anti-inflammatory and wound-resolution microglial phenotypes (Chatterjee et al., 2014), which reduces a person’s ability to recovery from SCI (Fenn et al., 2014). Therefore, the age factor exacerbates the inflammatory pattern increasing susceptibility to inflammatory diseases such as diabetes, cardiovascular disease, rheumatoid arthritis, respiratory infections, and gastrointestinal-renal disorders. In conclusion, aging itself does not lead to inflammatory diseases yet aging makes one less tolerant to fluctuations in homeostasis; therefore, aging persons with SCI are more likely to succumb to inflammatory diseases which leads to greater reductions in hippocampal neurogenesis.

Aging itself does lead to a great variety of cellular processes such as more senescent cells (Rea et al., 2018) and an age-dependent decline in autophagy efficiency, increasing the probability of cytoplasmic protein aggregation leading to a higher prevalence of neurodegenerative diseases (Nah et al., 2015). Furthermore, aging decreases mitochondrial enzyme efficiency, respiratory capacity, and phosphocreatine recovery time (Sun N. et al., 2016). Mitochondria undergo morphological changes during neurogenesis, shifting from glycolysis to oxidative phosphorylation as the newly differentiated neurons lose glycolysis associated genes (Shin et al., 2015). The impairment of mitochondrial bioenergetics prohibits the differentiation of NPCs into neurons (Lorenz et al., 2017), reduces NSC/NPC proliferation and newborn hippocampal neurons, leading to cognitive deficits (Voloboueva and Giffard, 2011; Khacho et al., 2017). SCI also impairs mitochondrial bioenergetics in the spinal cord (Sullivan et al., 2007). It has yet to be determined if SCI induces mitochondrial impairments in the hippocampus. Since both the mental health of an individual and newborn neurons are reliant on oxidative phosphorylation, aging SCI patients often endure many neurological and psychiatric symptoms at higher rates (Son and Han, 2018). We hypothesize that the age-induced decline in mitochondrial function and efficiency is compounded by the effects of SCI induced mitochondrial dysfunction leading to greater reductions in adult hippocampal neurogenesis resulting in higher rates of cognitive decline during chronic SCI. This theory remains to be tested; however, if correct, targeting mitochondrial activity will potentially enhance neurogenesis and reduce the SCI-induced memory and cognitive dysfunctions.

## Potential Mechanisms Modulating Neurogenesis After Chronic SCI

There currently is no consensus on the exact mechanism for the reduction of neurogenesis after SCI and very few articles investigated this matter. Some potential pathways altering neurogenesis in other diseases and associated with SCI, include the increased pro-inflammatory cytokine production, glial cell activation and reduced autophagy. Here, we will discuss SCI-induced changes that have potential to modulate hippocampal neurogenesis.

### Impact of Pro-inflammatory Cytokines Associated With SCI on Neurogenesis

Spinal Cord Injury is associated with increased pro-inflammatory cytokine expression in the brain, such as TNF-α, IL-6 (Wu et al., 2014), and IL-1β (Jure et al., 2020), which have potential to reduce adult hippocampal neurogenesis. In numerous pathologies and diseases leading to a reduction in hippocampal neurogenesis, pro-inflammatory cytokines have been implicated as the main culprit. Chronic IL-1β expression leads to neurodegeneration, impaired memory, and EAE (Ferrari et al., 2006; Shaftel et al., 2008; Hewett et al., 2012; Lin and Edelson, 2017) by activating STAT3-induced astrogliosis inhibiting neuronal differentiation (Chen et al., 2013). While exercise is a potent activator of hippocampal neurogenesis (Rhodes et al., 2003; Liu and Nusslock, 2018; Trinchero et al., 2019), IL-1β can prevent the exercise-induced rescue of hippocampal neurogenesis (Wu et al., 2012). Sustained overexpression of IL-1β in the hippocampus directly impedes hippocampal neurogenesis (Wu et al., 2013; Pawley et al., 2020) and mediates the decrease of adult hippocampal neurogenesis after ethanol exposure (Crews, 2012).

IL-1β expression is also increased by GSK-3β which inhibits Wnt signaling (Badimon et al., 2019) and orphan nuclear receptor tailless homolog (TLX) expression involved in NSC maintenance and fate determination (Zhao et al., 2009; Islam and Zhang, 2015). GSK-3β inhibition, which in of itself increases hippocampal neurogenesis (Guo et al., 2012; Morales-Garcia et al., 2012), significantly reduces the effects of IL-1β mediated inhibition of TLX which also increases hippocampal neurogenesis (Green and Nolan, 2012; Ryan et al., 2013). The deletion of TLX not only decreases hippocampal neurogenesis (Kozareva et al., 2018), but may also increase susceptibility to microglial-induced hippocampal inflammation (Kozareva et al., 2019) which can further impede neurogenesis. GSK-3β is also an autophagy inhibitor mediated by mammalian target of rapamycin (mTOR) complex 1 (Mancinelli et al., 2017) and its inhibition successfully increased autophagy (Weikel et al., 2016). Therefore, GSK-3β is a vital target for recovery, as impairing autophagy leads to a dysfunctional immune system (Wang et al., 2019; Pehote and Vij, 2020).

Chronic NF-κB activation, mediated by neuronal NOS (Zhu et al., 2018), suppresses hippocampal neurogenesis (Koo et al., 2010). Chronic IL-6 has also shown to impair hippocampal neurogenesis (Vallières et al., 2002) although its effects are time- and context-dependent. During development, bursts of IL-6 production increase the NSC population while long-term IL-6 exposure reverses its effects on NSCs (Storer et al., 2018). The dramatic increase in IL-6 production after focal cerebral ischemia is hypothesized to prevent neuronal death (Loddick et al., 1998). TNF-α activates NF-κB signaling, decreasing NSC proliferation (Chen and Palmer, 2013) and hippocampal neurogenesis (Iosif et al., 2006). TNF-α expression also significantly reduces the neurite length and branching points of neurons (Neumann et al., 2002). Therefore, prolonged exposure to pro-inflammatory cytokines induced by SCI is likely to inhibit different stages of the hippocampal neurogenesis. Consequently, each cytokine might reduce hippocampal neurogenesis differently, and therefore impact neurological functions differently. It will be interesting to determine the exact mechanisms of each cytokines, and the precise stage of neurogenesis they might interfere with.

### Hippocampal Microglia and Neurogenesis

Microglia are the resident immune cells of the CNS, responsible for phagocytosis, nurturing neural cells, and are neuroprotective during infections and injury (Rock et al., 2004; Lenz and Nelson, 2018). Just 1-week post SCI, the number of activated microglia in the injury site increases ∼9-fold and remains elevated by ∼4-fold 4-weeks post injury (Zhou et al., 2018). In humans, increased activation of microglia has been found in the injury site months following SCI (Fleming et al., 2006). In the hippocampus, microglia are essential for hippocampal neurogenesis and neuronal differentiation (Appel et al., 2018). Microglial depletion after SCI alleviated depression, cognitive dysfunction, and neuron death in the hippocampus (Li et al., 2020b). Yet, activated microglia during chronic inflammation secrete cytokines to induce apoptosis before phagocytizing hippocampal neurons (Ekdahl et al., 2003), inhibiting neuronal differentiation and favoring astrocytic differentiation (Diaz-Aparicio et al., 2020). Hippocampal microglia activation is known to impair NPC proliferation and hippocampal neurogenesis in non-SCI models (Stefani et al., 2018). After SCI, the number of activated and total microglia in the hippocampus of rats increase in a severity-dependent manner at 60 dpi while the number of ramified microglial remain unchanged (Jure et al., 2017). However in mice, at 50 dpi, IL-1β, IL-18 and TNF-α expression are all significantly increased in the hippocampus while the number of microglial cells in the hippocampus are reduced (Jure et al., 2020). Possible reasonings for the increase in cytokine production in mice during the decrease in microglial cells are that these cytokines are being produced by other neural cells, such as astrocytes (Cekanaviciute and Buckwalter, 2016), or the hippocampal microglia are overactive and produce more cytokines locally, or a combination of the two. There are several reasons for the discrepancy between Jure’s (2017, 2020) study. Jure’s (2020) study only analyzed the brains of mice with moderate SCI yet Jure’s (2017) study analyzed the brains of rats with SCI of different severities. As previously mentioned, mice and rats have different inflammatory responses to SCI (Sroga et al., 2003). The moderate injury in the 2020 study might not have evoked enough of a response to elevate the number of microglia in the hippocampus of mice at 50 dpi. The 2020 study used Iba-1 (cytoplasm protein) marker in contrary to the Ox42 (membrane protein) marker used in the 2017 study. The 2017 study is expected to see a larger increase in microglia compared to the 2020 study even though microglial response between studies should not be compared due to changes in specie models which evoke different inflammatory responses (Sroga et al., 2003). Even at 90 dpi, IL-6, TNF-α, and iNOS expression remains elevated in the hippocampus. IL-10 expression in the hippocampus is reduced at 8 dpi while being elevated at 90 dpi (Wu et al., 2014); although anti-inflammatory, IL-10 reduces neuron differentiation and neurogenesis (Perez-Asensio et al., 2013).

Chronic overactivation of microglia in the hippocampus might also be due to the increased prevalence of infections in persons with SCI. Bacterial infections activating the TLR4 receptor on microglia through NF-κB signaling activate microglial production of TNF-α, IL-1β, iNOS, ROS, and NO, increasing neuroinflammation (Fernandez-Lizarbe et al., 2009; Yao et al., 2013). In short duration, NO promotes hippocampal NSC proliferation, but long-term exposure to NO is detrimental to newborn neuron survival (Carreira et al., 2015). ROS are generated in small quantities during normal adult hippocampal neurogenesis (Walton et al., 2012), but can be toxic to neurons in large quantities, causing DNA damage (Hemnani and Parihar, 1998) and increasing susceptibility to neurodegenerative disorders (Gilgun-Sherki et al., 2001). Microglia are activated for neuroprotection, yet, similar to other inflammatory pathways, overactivation leads to aberrant behavior resulting in declined hippocampal neurogenesis and is associated with cognitive deficits (Wu et al., 2014).

Microglia also have a great impact on the activity and proliferation of other neural cells, such as astrocytes, regardless of the pathology or location. Chronically activated microglia mediate astrogliosis (Zhang et al., 2010), a transformation of astrocytes to an activated state (Sofroniew, 2014). Microglial production of lymphotoxin, TNF, and IL-6 are mitogenic for astrocytes (Barna et al., 1990; Selmaj et al., 1990), while production of prostaglandin (PG)D_2_ and IL-1β mediates astrogliosis (Herx and Yong, 2001; Mohri et al., 2006). The role of astrocytes in hippocampal neurogenesis is far-reaching. Astrocytes assist in integrating newborn hippocampal neurons into the existing neuron network (Krzisch et al., 2015) and are essential in preserving the integrity of the blood brain barrier (Lien et al., 2012). After SCI, the population of reactive astrocytes is increased across the hippocampus at both 7 and 50 dpi (Jure et al., 2020). It is not clear if this astrocytic response is the cause or the consequence of the increase in the microglial activation and if the local increase of pro-inflammatory molecules from astrocytes and microglia in the hippocampus are reducing neurogenesis. Indeed, an alternative hypothesis would be that molecules transported systemically from the spinal lesion site to the hippocampus directly reduce neurogenesis and increase the activation of astrocytes and microglia. As mentioned above, another possibility is that systemic inflammation induces hippocampal microglia activation and astrogliosis, which in turn secrete local molecules reducing hippocampal neurogenesis. However, whether these beneficial effects are the direct results of microglia depletion in the hippocampus, or indirectly through changes in astrocyte reactivity remains to be determined.

### Alteration of Debris Removal, Autophagy, and Intracellular Processes After Chronic SCI

Autoimmunity is a significant threat to longevity, causing aberrant and chronic inflammatory responses. After SCI, microglia participate in the phagocytosis of cell debris (Green et al., 2019) and prune neurons through NF-κB activated astroglial release of complement C3 (Stevens et al., 2007; Chu et al., 2010). Defective clearance of apoptotic cells in any region has been attributed to the progression of autoimmune disorders (Schwab et al., 2014). The debris from the apoptotic cells acts as a survival signal for B cells while the left over nucleic acids mimic viral infections (Munoz et al., 2010). This aberrant B-cell activation leads to dysregulation of antibody production, and autoimmune reactions to CNS proteins (Ankeny et al., 2006). Reduction of debris removal in the spinal cord after chronic SCI is likely to participate in the chronic inflammation state, indirectly reducing hippocampal neurogenesis. Alternatively, if SCI induces debris accumulation in the hippocampus during chronic SCI, it might participate in the higher prevalence of neurodegeneration seen in persons with SCI (Amanat et al., 2019). However, the increase in the debris accumulation in the hippocampus after SCI and respective mechanisms remain to be demonstrated.

Autophagy flux is the process of degradation of cytoplasmic proteins and organelles (du Toit et al., 2018) which is vital for proper oligodendrocyte function (Bankston et al., 2019), minimizing neuronal damage (Tang et al., 2014), and essential for improving locomotor function (Saraswat Ohri et al., 2018). Autophagosomes’ ability to degrade LPS containing pathogens (Bah and Vergne, 2017; Hagio-Izaki et al., 2018) and decrease IL-1β secretion (Harris et al., 2011; Iula et al., 2018) should modulate the activity of the innate immune system. Although autophagosomes accumulate after SCI (Zhang et al., 2014; Muñoz-Galdeano et al., 2018), autophagy flux in the spinal cord is impaired (Liu S. et al., 2018). Impaired autophagy flux, which can induce neurodegeneration (Hara et al., 2006; Komatsu et al., 2006; Liu S. et al., 2015), has been implicated to cause inflammation and autoimmune disorders in many disease models (Hsieh et al., 2017; Lambelet et al., 2018; Vij et al., 2018), especially in neurogenerative diseases (Nixon, 2013). Using the FIP200 knockdown model for studying autophagy, Wang et al. found autophagy improves NSC survival, proliferation and differentiation into neurons, assists with NSC maintenance, and overall increases adult neurogenesis (Wang et al., 2013). Further experimentation is required to determine how SCI directly influences autophagy in the hippocampus.

Spinal Cord Injury patients, on average 28 years after injury, displayed a 525% increase in IL-6, 115.9% increase in circulating vascular adhesion molecule (sVCAM)-1, and 44.4%increase in endothelin-1 (Wang et al., 2007). This may be due to SCI-induced alterations in gene expression, as patients over a year after injury had natural killer (NK) related genes under expressed while TLR, TNF-α, epidermal growth factor (EGF), IL, insulin, CXCR4, and PDGF pathways were over expressed (da Silva Alves et al., 2013; Herman et al., 2018). These significant changes may be due to SCI-induced sympathetic nervous system dysfunction (Campagnolo et al., 1994) or through a perpetual cycle of chronic inflammation, infections, and dysfunctional immune system. NK and its constituent, 12/15-lipoxygenase, responsible for destroying and phagocytizing viral particles, are significantly downregulated even several years after SCI (Campagnolo et al., 2008; Uderhardt et al., 2012). NLRP3 inflammasomes and associated pro-inflammatory pathways are further activated, followed by dysfunctional autophagosomes increasing the presence of bacterial LPS (Mouasni et al., 2019) while the prevalence of infections increases (Evans et al., 2008; Tofte et al., 2017). This predicament creates a simultaneous increase in inflammatory cytokine production and infection rate. Overactivation of the NLRP3 inflammasome pathway can be harmful as it induces microglia mediated IL-1β secretion (Pan et al., 2014) which is already elevated due to the lack of inhibition from the dysfunctional autophagosomes and increased LPS presence. This is compounded by diminished response to newer threats as antibody production and CD8 + T cell response are both impaired, increasing the probability of people with SCI succumbing to otherwise non-fatal infections (Bracchi-Ricard et al., 2016). This creates a positive feedback loop where autophagy disfunction increases the prevalence of both autoimmune inducing debris formation and infection inducing pathogens, both increasing pro-inflammatory pathways which further increases the prevalence of inflammation inducing pathogenesis; altogether, increasing the probability of impaired hippocampal neurogenesis. Altogether, changes in debris removal, autophagy, and intracellular processes in the chronic phases of SCI, either at the spinal cord lesion site or in the hippocampus, via systemic or direct effects on the neurogenic niche, may play a complex and important role in the modulation of neurogenesis and must be further evaluated in the context of SCI.

### SCI-Induced Endoplasmic Reticulum Stress in the Hippocampus and Neurogenesis

Endoplasmic reticulum (ER) is an essential part of all cells for sorting, packaging, and modifying proteins vital for cell survival. ER stress is associated with several neurodegenerative diseases, ischemia, diabetes, bipolar disorder and can lead to cell death (Raghubir et al., 2011). GRP78, a marker for ER stress (Lee, 2005), is still elevated in the hippocampus 4 months post SCI in a linear relationship to the severity of the SCI (Wu et al., 2016) implicating SCI causes chronic ER stress. Chronic ER stress has been shown to elevate inflammatory markers, CXCL3 and CXCL10 (Ha et al., 2015), induce neuronal death (Hood et al., 2018), reduce NSC proliferation (Yang et al., 2020), and impair autophagy (Ogata et al., 2006). JNK, which is dysregulated by chronic inflammation (Lessel et al., 2017), mediates the ER stress-induced impairment of autophagy, and inhibits the maturation of newborn hippocampal neurons (Mohammad et al., 2018). CCL21, which is also elevated 4 months post SCI in the hippocampus (Wu et al., 2016), is mediated by CXCL3 alongside CXCL10 to activate microglia (Rappert et al., 2002; Lessel et al., 2017). The continuous CCL21 accumulation in the hippocampus after SCI may lead to chronic microglial activation and is hypothesized to be the reason there is chronic microglial activation in areas distant from the injury site (de Jong et al., 2005) such as the hippocampus. ER stress and associated processes in hippocampal cells can be one mechanism of reduced hippocampal neurogenesis during chronic SCI. The demonstration that SCI-induced ER stress directly reduces neurogenesis has not been established yet.

### Conclusion

Spinal Cord Injury-induced inflammation, whether directly or indirectly induced, has a tremendous impact on longevity and quality of life of persons living with SCI. Inflammation also has vital roles in maintaining a defense system and helping repair and regenerate tissue. The intensity, timing and duration of the inflammatory response is crucial for determining its all-encompassing effects. SCI leads to increased prevalence of many secondary pathologies, such as impaired autophagy, ER stress, chronic pro-inflammatory cytokine production, infections and many others. Consequently, the constantly activated endogenous inflammatory system starts behaving aberrantly in a self-destructive manner which not only decreases the lifespan of persons with SCI, but also substantially reduces their quality of life. These pathologies lead to a perpetual positive feedback loop which generate further complications such as neurodegenerative, gastrointestinal and cardiometabolic disorders which compound the effects of the initial pathologies. As such, many researchers are targeting the immune system. Modulating inflammation appropriately has therapeutic potential; although, modulation has also negatively impacted functional locomotor recovery when incorporated incorrectly (Beck et al., 2010). Therefore, it is vital to holistically study and understand the long-term impact of immune modulation before reaching clinical trials for anti-inflammatory therapeutics.

## Therapeutic Options

We will discuss below potential options to treat or prevent the SCI-induced reduction in hippocampal neurogenesis and mitigate cognitive dysfunction and memory impairments in people with SCI. The treatment options discussed herein are intended to either act directly on hippocampal processes or indirectly in a holistic manner to counteract the many secondary pathologies induced by inflammation (Figure 2). Many of the treatment options discussed herein have not been tested to increase neurogenesis in preclinical SCI models or persons living with SCI. Therefore, the efficacy of said treatments are speculative based on their mechanism of action.

### Exercise

Exercise has been shown to enhance hippocampal neurogenesis (Rhodes et al., 2003) and increase memory capacity (Loprinzi et al., 2018). Therefore, exercise is a potential treatment option alleviating the reduction of neurogenesis after SCI via increased hippocampal BDNF levels (Hötting et al., 2016), decreased pro-inflammatory cytokine expression, and increased anti-inflammatory cytokine expression (Petersen and Pedersen, 2005; Flynn et al., 2007; da Silva Alves et al., 2013). As previously mentioned, exercise can also increase hippocampal neurogenesis through epinephrine (Ahmadiasl et al., 2003), therefore, impaired epinephrine production (such as in the context of SCI) may decrease the anti-inflammatory effects of exercise. Additionally, exercise is known to reduce susceptibility to depression (Stanton and Reaburn, 2014) and improve psychological wellbeing (Callaghan et al., 2011). Exercising after SCI is a challenge and may not always be an option especially for persons with severe or high cervical injuries. It would be of great clinical interest to test whether new technologies can stimulate muscles to provide some of the benefits associated with exercise (Ragnarsson, 2008). Alternatively, passive range of motion exercises with a partner/caregiver may produce the same impact as regular exercise; although, its effects on inflammation and hippocampal neurogenesis have yet to be elucidated.

### Nutrition

Dietary choices have also shown to have a profound impact on the overall inflammatory state of an individual, with or without SCI. As discussed above, we hypothesized that reducing inflammation after SCI is an attractive strategy to target hippocampal neurogenesis. However, how an anti-inflammatory diet might slow down or reverse hippocampal neurogenesis after SCI has yet to be test. A 12-week anti-inflammatory diet (foods with anti-inflammatory effects such as berries, fish, and broccoli) not only decreased pro-inflammatory cytokines, IL-2, and IFN-γ, but also reduced SCI-induced neuropathic pain (Allison et al., 2016). A high-protein diet given to people living with SCI decreases TNF-α levels with improved insulin sensitivity (Li Y. et al., 2018) which increases hippocampal neurogenesis and plasticity (Spinelli et al., 2019). Moreover, mice with SCI on the ketogenic diet (high fat, moderate protein, low carbohydrate) present a reduction in NF-κB, TNF-α, IL-1β, IFN-γ, and oxidative stress levels, activation of Nrf2 (Lu et al., 2018), and improved forelimb motor control (Streijger et al., 2013). Nrf2 activation has anti-oxidative effects vital for aging brains to maintain their rate of hippocampal neurogenesis, specifically, inducing NSC/NPC proliferation. Nrf2 also increases survival and integration of transplanted NSC/NPC (Robledinos-Antón et al., 2017; Ray et al., 2018). In humans, the increased uptake of ω-3 fatty acids mitigates the age-induced cognitive decline (van Gelder et al., 2007). Vitamin B/D/E, flavonoids, and curcumin are also of great interest to target cognitive decline (Gómez-Pinilla, 2008). Specific studies are necessary to demonstrate the direct influence of diet on hippocampal neurogenesis and cognition in persons with chronic SCI.

### Alterations of the Microbiome

The use of microbiome alterations for therapeutic purposes has become a rapidly evolving field as gut inflammasomes can influence brain pathology through the gut-brain axis (Rutsch et al., 2020). Recent advancements suggest microbiome alterations have the potential to help persons with SCI by improving locomotor function and immune health (Kigerl et al., 2018). Bacteria are known to directly influence not just our inflammatory pathways, but also neurogenesis and neurological disorders. Therefore, reducing systemic inflammation through modulation of the microbiome might be a strategy to increase neurogenesis. A recent study showed that alteration of the gut microbiome alters hippocampal neurogenesis in both an age- and sex-dependent manner, with young male germ-free mice showing decreased hippocampal neurogenesis and increased neuron apoptosis (Scott et al., 2020). Gut microbiome from 2-year-old mice transplanted into 6-week-old germ-free mice increased the number of newborn hippocampal neurons and local BDNF expression in the short-term while the transplantation of microbiome from 6-week-old mice had no effect (Kundu et al., 2019). It is unclear if this effect is induced by acute inflammation or as a response to a pathology caused by the microbiome transplantation from older mice. Therefore, the contents of, sensitivity to, and effects of microbiome transplantation are age dependent. Specific bacterial strains have shown unique therapeutic potential, although none of the following strains have been tested in pre-clinical models of SCI as way to modulate inflammation, recovery, or hippocampal neurogenesis. *Enterococcus faecalis*, although inflammasome inducing and frequently found in the pressure ulcers of SCI patients, has shown therapeutic potential to reverse the suppression of inflammatory bowel disease-induced impairment of hippocampal neurogenesis (Takahashi et al., 2019). Another strain from the same genus, *Enterococcus durans*, significantly decreases IL-1β, IL-17, IFN-γ, IL-6, and CXCL1 expression in the Peyer’s patches of treated mice while supporting local *Faecalibacterium prausnitzii* populations (Carasi et al., 2017). *Faecalibacterium prausnitzii* decreases TNF-α and IL-12 expression while increasing IL-10 expression in the colons of mice with Crohn’s disease (Sokol et al., 2008). *Bifidobacterium breve* has shown promise in increasing hippocampal BDNF levels (O’Sullivan et al., 2011) and suppressing hippocampal inflammation (Kobayashi et al., 2017). A combinational therapy of *Lactobacillus helveticus* and *Bifidobacterium longum* attenuates stress-induced suppression of hippocampal neurogenesis (Ait-Belgnaoui et al., 2014). *Lactobacillus rhamnosus* modulates GABA receptor mRNA expression and provides protection against stress (Bravo et al., 2011; McVey Neufeld et al., 2019). Heat−killed *Lactobacillus brevis* enhances hippocampal neuron survival and improves memory (Ishikawa et al., 2019). Lastly, *Lactobacillus plantarum* increases BDNF, serotonin, serotonin transporter, and neurotrophin expression in the brain while elevating intestinal serotonin concentrations with potential antidepressant properties (Choi et al., 2019; Ranuh et al., 2019; Rudzki et al., 2019). Interestingly, fecal transplantation in rats after SCI reduces gut dysbiosis, and the development of anxiety and depression (Schmidt et al., 2020). Both depression and anxiety are associated with the reduction in hippocampal neurogenesis in non-SCI models (Revest et al., 2009; Eisch and Petrik, 2012; Hill et al., 2015). Therefore, an attractive theory would be that fecal transplantation reduces the loss of neurogenesis, via changes in inflammation, ameliorating anxiety/depression in rats with SCI. Altogether, these data suggest a complex relationship between microbiome, inflammation, and modulation of hippocampal neurogenesis. Personalized medicine is likely to be appropriate here to create special blends of probiotic supplements based on the beneficial strains missing from the patient’s gut microbiome. While direct applications to SCI models and hippocampal neurogenesis remain to be tested, the translational potential of probiotic treatment after SCI is of interest to potentially reduce systemic inflammation and ameliorate neurogenesis, memory, and cognition.

### Anti-inflammatory Therapeutics

As discussed, we hypothesize that chronic inflammation after SCI is responsible for the reduction in neurogenesis. Suppressing aberrant inflammatory processes during chronic SCI is vital to improving longevity and overall health as many of the secondary pathologies following SCI are chronic inflammatory diseases which generally have a negative impact on hippocampal neurogenesis. Several anti-inflammatory therapeutics have been shown to improve hippocampal neurogenesis and would therefore be potential candidates to minimize secondary pathologies while mitigating the decline in hippocampal neurogenesis. Methylprednisolone perturbs the cytotoxic effects of pro-inflammatory cytokines, such as TNF-α and IL-6, and impedes the activation of T cells while increasing the apoptosis rate of activated immune cells (Sloka and Stefanelli, 2005). After transient cerebral ischemia, acute methylprednisolone treatment increases both the number and migration rate of newborn neurons (Jing et al., 2012) which are essential for the survival and integration of newborn neurons. However, based on 13 clinical studies, acute methylprednisolone treatment has less than a modest effect on neurological function (Hugenholtz et al., 2002) while increasing an SCI patient’s risk of pneumonia 2.6-fold (Gerndt et al., 1997). Erythropoietin, which hinders the actions of pro-inflammatory cytokines (Peng et al., 2020), not only increases locomotor recovery after SCI, but also the number of newborn hippocampal neurons (Zhang et al., 2018). Acute treatment with minocycline, an anti-inflammatory and anti-biotic agent, provides neuroprotection and enhances behavioral recovery, hindlimb function, and strength in mice with SCI (Wells et al., 2003; Elewa et al., 2006). Minocycline also mitigates age-induced spatial learning deficits and increases the number of newborn neurons in the hippocampus of non-injured 3-month old mice (Kohman et al., 2013). Chondroitinase ABC, which degrades pro-inflammatory CSPGs, promotes plasticity in the spinal cord (Barritt et al., 2006) and brain (Chen et al., 2014), augments locomotor recovery following SCI (García-Alías et al., 2011), is neuroprotective (Chen et al., 2014), and prevents social defeat–induced persistent stress memory loss (Riga et al., 2017), all of which can positively impacts hippocampal neurogenesis. Importantly, any treatment improving locomotor function will increase physical activity, thereby likely improving hippocampal neurogenesis and cognitive function. Notably, proper functioning inflammatory systems, especially during development, are vital components of neurogenesis (Storer et al., 2018). Therefore, anti-inflammatory therapeutics must be given to younger patients with extreme caution. All of these anti-inflammatory therapeutic options show promise in enhancing hippocampal function or related cognitive function. None of the SCI patients have received these treatments to directly enhance their cognitive capacity or hippocampal neurogenesis, therefore, more experimentation is required in the context of SCI.

### Metabolism and Mitochondria

There is evidence of mitochondrial dysfunction in the spinal cord, with impaired mitochondrial bioenergetics as early as 12 h after SCI (Sullivan et al., 2007). Mitochondria modulate synaptogenesis, fuel hippocampal neurogenesis and assist with cognitive development and memory formation (Arrázola et al., 2019; Khacho et al., 2019). Inducing mitochondrial dysfunction inhibits NSC growth and differentiation into neurons and impairs immature hippocampal neuron maturation (Agnihotri et al., 2017). Therefore, cellular energy capacity can be a limiting factor in hippocampal neurogenesis. Mitochondria have recently been a target of interest for repair strategies, although its specific effects on hippocampal neurogenesis in the context of SCI have yet to be tested. Lithium increases electron transport chain activity, theoretically increasing respiratory capacity of mitochondria. Lithium is hypothesized to reverse the impaired energy generation in the brains’ of bipolar disorder patients (Maurer et al., 2009). Lithium tested on animal SCI models showed inhibition of GSK-3β while doubling the effects of chondroitinase ABC-induced increase in axon regeneration (Yick et al., 2004). Therefore, increasing mitochondrial bioenergetics does have potential in impeding chronic inflammation and improving locomotor function. As previously noted, inhibition of GSK-3β can increase hippocampal neurogenesis (Guo et al., 2012; Morales-Garcia et al., 2012). Cyclosporin A, an immunosuppressant that enhances mitochondrial function through inhibition of mitochondrial permeability transition pore (Scholpa and Schnellmann, 2017), enhances hippocampal neurogenesis by increasing NPC survival and number of newborn neurons (Hunt et al., 2010; Chow and Morshead, 2016).

However, mitochondrial function is not analogous to immunosuppression; mitochondria are integral parts of an active immune system from activating inflammation during infection to pro-inflammatory signaling (Missiroli et al., 2020). Intravenous mitochondria injections not only increased endurance in mice, but also increased energy production and ATP levels in the brain while decreasing neural ROS levels in a Parkinson’s disease model (Shi et al., 2017); ROS generally decreases neuron survival (Drukarch et al., 1998). Recently, mitochondrial transplantation in the spinal cord has been tested in an SCI model, and despite not promoting functional recovery, showed encouraging results for maintaining bioenergetics (Gollihue et al., 2018). Mitochondrial transplantation in a stroke model can mitigate neuron death and ROS and increase neurogenesis in the subventricular zone (Zhang et al., 2019). Altogether, mitochondria-based therapeutics show promise for promoting hippocampal neurogenesis although its mechanism of action is complex and must be further elucidated in the context of SCI.

### Extracellular Vesicles

Another promising therapeutic option for increasing hippocampal neurogenesis, reducing inflammation, and improving locomotor function is the use of mesenchymal stem cell derived extracellular vesicles (MSC-EV). MSC-EVs improve functional locomotor recovery after SCI via suppression of the activation of neurotoxic reactive astrocytes and shift microglial polarization from pro-inflammatory to anti-inflammatory profiles (Liu W. et al., 2018; Liu et al., 2020). MSC-EVs effectively decrease astrocyte reactivity and microglial activation after TBI as well (Yang et al., 2017). MSC-EVs mitigate apoptosis of neurons and increase locomotor recovery through the Wnt/β-catenin signaling pathway (Li et al., 2019). The Wnt/β-catenin signaling pathway is essential to hippocampal neurogenesis as it supports the proliferation of NSC/NPC and the maturation and integration of newborn neurons (Zhang et al., 2011; Qu et al., 2013). MSC-EVs promote hippocampal neurogenesis (Reza-Zaldivar et al., 2019; Chen et al., 2020), prevent memory dysfunction after status epilepticus (Long et al., 2017), and improve cognitive impairments in diabetes (Nakano et al., 2016) and Alzheimer’s disease (Nakano et al., 2020) models. MSC-EVs can even be administered intranasally to target forebrain and hippocampal neurons while preserving functional activity preventing undesirable delivery to other regions of the CNS (Kodali et al., 2019). MSC-EVs are currently in a phase 2 clinical trial in stroke patients (Yazdani, 2019); testing in humans in the context of SCI remains to be performed.

## Alternative Pathway for SCI-Induced Modulation of Hippocampal Neurogenesis

Although many of the hippocampal changes induced by inflammation is hypothesized to be caused by systemic changes, the hippocampus might also interact with motor neurons possibly providing an alternative pathway for SCI-induced modulation of hippocampal neurogenesis. This hypothesis has yet to be evaluated. The axons of cortical layer V pyramidal cells descend down the corticospinal tract to control locomotion (Fitzpatrick, 2001). SCI-induced damage to these axons causes degeneration (Mittal et al., 2016; Hassannejad et al., 2019) which can modify the overall health of the cortical neurons and may eventually lead to neuronal death (Hu et al., 2012). The neocortex interacts with the hippocampus through axonal projections into the perirhinal cortex (van Strien et al., 2009) which projects to the entorhinal cortex (de Curtis and Paré, 2004). The entorhinal cortex then projects neurons onto area CA1 and the subiculum of the hippocampus (Witter et al., 2017). Neurons from the entorhinal cortex also project axons to the dentate gyrus (Andersen et al., 1969; van Groen et al., 2003). The direct pathway connecting the upper motor neurons to the hippocampus has yet to be fully elucidated. We hypothesize that the cellular responses of the cortical neurons after SCI, the changes in their connectivity and plasticity in the brain, and eventually their death, might influence hippocampal neurogenesis. Furthermore, the voluntary movement of limbs does activate the hippocampus even when learning or memory are not required (Burman, 2019), therefore, there is evidence for constant cross-talk between these regions. Nonetheless, current research has not determined the direct physical connection or communication between the hippocampus and motor cortex and how degeneration in one area can affect the other.

## Conclusion

Beyond the evident effect of causing paralysis, spinal cord injuries induce a plethora of health complications due to the dysfunction of peripheral organs. These include, but are not limited to, gastrointestinal, renal, respiratory, hepatic, and cardiometabolic issues. In addition, new evidence demonstrates that SCI also leads to cognitive and memory impairments and neurodegenerative and psychological disorders. One possible reason may be the SCI-induced accelerated decline in the rate of hippocampal neurogenesis compared to identically aged able-bodied individuals. This decline in hippocampal neurogenesis might be caused by the high rate of infections present in people living with SCI, detrimental effects of local and systemic chronic inflammation, hormonal imbalance, autophagy flux and ER impairments, or dysfunction of microglia and astrocytes in the hippocampus. A better understanding of the mechanisms of action of the SCI-induced reduction of neurogenesis at the cellular and molecular level is necessary. Current research is showing promising results in battling against chronic inflammation and neurodegeneration. Diet, exercise, alteration of the microbiome, and anti-inflammatory compounds are therapeutic tools that might be of interest in promoting neurogenesis and reducing SCI-induced neurological disorders. Yet their efficacy and direct effects on hippocampal neurogenesis after SCI remain to be tested. Furthermore, the mechanism of which SCI induces these neurological disorders, and the therapeutic benefits of augmenting hippocampal neurogenesis will have to be determined. This will require a collaboration between researchers, clinicians, and chemists as well as day-to-day caregivers, dieticians, and kinesiologists to develop and test new techniques, compounds, and procedures to help alleviate the harmful effects of chronic inflammation on neurological function while restoring proper immune function to mitigate further secondary pathologies.

## Author Contributions

AS and CG contributed equally to the work and approved it for publication.

## Conflict of Interest

The authors declare that the research was conducted in the absence of any commercial or financial relationships that could be construed as a potential conflict of interest.
